# Muscle stem cells at a glance

**DOI:** 10.1242/jcs.151209

**Published:** 2014-11-01

**Authors:** Yu Xin Wang, Nicolas A. Dumont, Michael A. Rudnicki

**Affiliations:** 1 Sprott Centre for Stem Cell Research, Ottawa Hospital Research Institute, Ottawa, ON K1H 8L6, Canada; 2 Faculty of Medicine, Department of Cellular and Molecular Medicine, University of Ottawa, Ottawa, ON K1H 8M5, Canada

**Keywords:** Regeneration, Satellite cells, Skeletal muscle, Stem cells, Therapy

## Abstract

Muscle stem cells facilitate the long-term regenerative capacity of skeletal muscle. This self-renewing population of satellite cells has only recently been defined through genetic and transplantation experiments. Although muscle stem cells remain in a dormant quiescent state in uninjured muscle, they are poised to activate and produce committed progeny. Unlike committed myogenic progenitor cells, the self-renewal capacity gives muscle stem cells the ability to engraft as satellite cells and capitulate long-term regeneration. Similar to other adult stem cells, understanding the molecular regulation of muscle stem cells has significant implications towards the development of pharmacological or cell-based therapies for muscle disorders. This Cell Science at a Glance article and accompanying poster will review satellite cell characteristics and therapeutic potential, and provide an overview of the muscle stem cell hallmarks: quiescence, self-renewal and commitment.

## Introduction

Skeletal muscle has the remarkable ability to regenerate from severe injuries. This regenerative capacity requires the activation and expansion of myogenic satellite cells (Wang and Rudnicki, 2012). Named for their relative anatomical position, satellite cells reside juxtaposed between a myofibre and its surrounding extracellular matrix (Mauro, 1961). Various degenerative or disease states affect the functional capacity of satellite cells and, consequently, impede muscle regeneration. Hence, research to understand satellite cell behaviour has gained significant interest in recent years.

**Figure:**
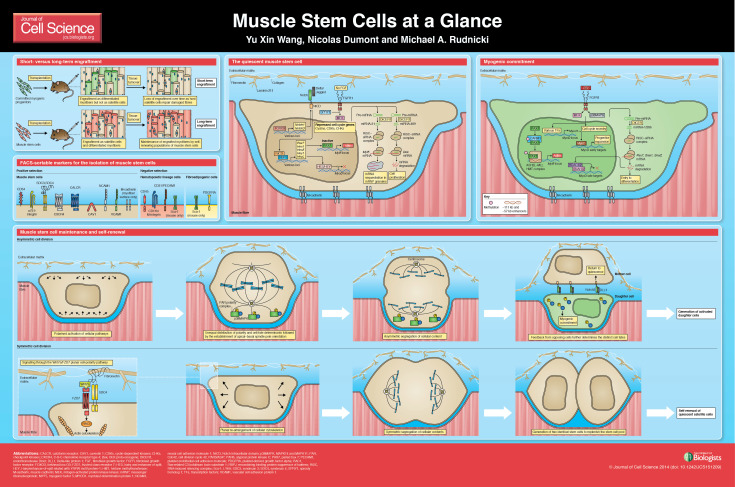


Since their discovery, satellite cells have been thought of as a strategic therapeutic target in treating acute muscle injuries and chronic diseases such as muscular dystrophies. Indeed, the ability of satellite cells and their progeny to fuse into the myofibre syncytium make them favourable vectors for delivering corrective gene therapy (Partridge et al., 1989). Unfortunately, cell-based therapies for muscle disorders are currently hindered by a lack of long-term engraftment. This has prompted the identification of specific sub-populations of satellite cells that are capable of self-renewal and engraftment as satellite cells in transplantation, coining the terms muscle stem cells or satellite stem cells. Here, we discuss satellite cell characteristics by providing an overview of the hallmarks of muscle stem cells, i.e. their quiescence, self-renewal capacity and lineage commitment. We also highlight their therapeutic potential.

## Finding muscle stem cells

The first engraftment assays were done by transplanting minced muscles − using H^3^-thymidine labelling or isoenzymes as markers − into wild-type muscles (Partridge and Sloper, 1977; Snow, 1978). These studies showed the formation of labelled regenerating myotubes in the host muscle, indicating that myogenic cells can efficiently fuse to allogenic fibres (Partridge et al., 1978). *In vitro* purification of satellite-cell-derived myoblasts followed by transplantation demonstrated that these cells are the sole contributors in the fusion with host myofibres (Lipton and Schultz, 1979). However, *in vitro* cultured myoblasts have low engraftment efficiency and exclusively differentiate into myofibres in transplants (Huard et al., 1992). Consequently, these engrafted myofibres are subject to tissue turnover and can only establish short-term engraftment.

With better purification methods and labelling for stem-cell-specific markers, recent transplantation studies have revealed sub-populations of freshly isolated satellite cells that can recapitulate the satellite cell compartment of recipient muscles (Collins et al., 2005; Kuang et al., 2007; Rocheteau et al., 2012; Sacco et al., 2008). These engrafted satellite stem cells give rise to committed myogenic cells while maintaining their stem cell identity through mechanisms of self-renewal. Importantly, transplanted bona fide muscle stem cells were preserved through multiple rounds of injuries, which is a prerequisite for a useful and long-term therapeutic approach (Sacco et al., 2008).

## Muscle stem cell markers

Satellite cells can be identified by the specific expression of certain proteins. Some markers are intracellular, such as the transcription factors PAX7 and the nuclear membrane proteins lamin A/C (LMNA) and emerin (EMD). Other markers are located at the cell membrane surface, such as syndecans 3 and 4 (SDC3 and SDC4), muscle (M)-cadherin, calcitonin receptor (CALCR), C-X-C chemokine receptor type 4 (CXCR4), calveolin 1 (CAV1), α7- and β1-integrins, neural cell adhesion molecule 1 (NCAM1), vascular cell adhesion molecule 1 (VCAM1) and CD34 (Fukada et al., 2007; Gnocchi et al., 2009) (see poster). Several laboratories have developed cell-sorting techniques to prospectively isolate satellite cells from muscle tissue. Most groups use a combination of positive selection for satellite cell surface markers, such as α7-integrin and CD34, and a negative selection for hematopoietic, fibrogenic lineages with antibodies against CD45, CD11b, CD31 and LY6A (also known as Sca-1) (Pasut et al., 2012). Other groups have raised antibodies against satellite-cell-specific antigens that are useful in isolating quiescent or activated satellite cells (Fukada et al., 2004).

Interestingly, variable expression of different markers, such as MYF5 and CD34, suggests the existence of different subpopulations of satellite cells (Beauchamp et al., 2000). Indeed, it has been demonstrated that, in quiescent muscles, ∼10% of satellite cells have never expressed MYF5, and that these cells possess self-renewal potential and long-term engraftment capacity (Kuang et al., 2007). These MYF5^−^ satellite cells represent a stem cell subpopulation that can give rise to MYF5^+^-committed satellite cells through asymmetric division.

Accordingly, dye-dilution studies that examine cell cycle kinetics by using labelling with PKH26 or BrdU showed that, in the activated state, satellite cells exhibit heterogeneous behaviour − with the majority of satellite cells undergoing fast division and the minority of cells undergoing slow division (Ono et al., 2012; Schultz, 1996). These slow-dividing satellite cells have long-term self-renewal ability and can divide asymmetrically − two hallmarks of stem cell behaviour. Label retention experiments by using BrdU confirmed that this subpopulation of satellite stem cells can maintain its original template DNA strands during cell division (Shinin et al., 2006). Consistently, a transgenic mouse model showed that, during regeneration, satellite cells that express higher levels of PAX7 (Pax7^Hi^) possess a lower metabolic rate and higher self-renewal ability (Rocheteau et al., 2012). The same authors demonstrated that, during division, Pax7^Hi^ cells can segregate their chromosomes asymmetrically in order to generate a distinct daughter cell, whereas cells with low PAX7 expression (Pax7^Lo^) segregate their DNA randomly. Altogether, these results indicate that satellite cells are a heterogeneous population that can be divided into two subpopulations: committed progenitor cells and muscle stem cells. The latter can divide asymmetrically in order to give rise to myogenic progenitors or can self-renew in order to maintain the pool of satellite cells. However, intrinsic differences between these subpopulations are still unclear and a practical marker to distinguish the subpopulations of satellite stem cells is still missing.

## The quiescent muscle stem cell

As other tissue-resident stem cells in the adult body, muscle stem cells remain quiescent during healthy resting periods (Cheung and Rando, 2013). In this G_0_ state, quiescent stem cells have a low metabolism and are more resistant to DNA damage. The quiescent state is required for the long-term maintenance of muscle stem cells. Loss of the capacity to remain quiescent generally leads to the precocious differentiation and loss of satellite cells over time. Indeed, long-term labelling experiments using transgenic doxycyclin-inducible H2B-GFP mice revealed that, under normal conditions, a proportion of PAX7^+^-satellite cells can remain quiescent for most of the lifetime of the animal and retain their full stem cell function despite changes in the muscle stem cell niche (Chakkalakal et al., 2012).

Quiescence of muscle stem cells is maintained by the combination of transcriptional repression of key cell cycle genes and high expression of cell cycle inhibitors. Notably, low expression levels of cyclins, cyclin-dependent kinases (CDKs) and checkpoint kinases (CHKs) in quiescent satellite cells prevent cell cycle progression (Fukada et al., 2007). Additionally, the expression of the CDK inhibitor p27/Kip1 (also known as CDKN1B) and the transcriptional silencer p130/Rbl2 (also known as RBL2) are required for the return to and maintenance of the G_0_ state (Cao et al., 2003). Correspondingly, muscle stem cells of germline *Cdkn1b*-knockout mice have a reduced capacity to self-renewal and their numbers decline over serial injury experiments (Chakkalakal et al., 2014).

The necessity for muscle stem cells to become rapidly activated in response to injury requires them to be poised to return to cell cycle and be primed to express downstream myogenic transcription factors. The expression of PAX7 not only demarcates the satellite cell pool but also specifies their myogenic lineage (Seale et al., 2000). Moreover, PAX7 expression is required to maintain the proper myogenic function of satellite cells throughout life (Günther et al., 2013; von Maltzahn et al., 2013). PAX7 is able to recruit the complex of the histone methyltransferases ASH2L and MLL (also known as KMT2A) to the *Myf5* locus and deposits permissive epigenetic marks to allow transcription (McKinnell et al., 2008). This activity is further regulated by the posttranslational modification of PAX7 by the histone-arginine methyltransferase CARM1 (Kawabe et al., 2012). In MYF5^−^ satellite stem cells, PAX7 is able to pre-emptively bind at the *Myf5* promoter region, but cannot recruit the ASH2L−MLL complex to trigger *Myf5* transcription. This creates a poised epigenetic environment that rapidly leads to the expression of myogenic transcription factors (see below).

Quiescent muscle stem cells express a variety of cellular receptors that facilitate signal transduction in order to reactivate cell cycle genes (see poster). Experiments that examined the early time points of satellite cells cultured on single myofibres established that fibroblast growth factor 2 (FGF2) is a key trigger for satellite cell activation (Yablonka-Reuveni et al., 1999). FGF2 is released by damaged myofibres and proliferating myoblasts, and sequestered by the extracellular matrix that makes up the muscle stem cell niche (Chakkalakal et al., 2012; DiMario et al., 1989; Hannon et al., 1996; Kästner et al., 2000). In the interest of maintaining long-term quiescence, FGF signalling is buffered in quiescent muscle stem cells by high levels of sprout homolog 1 (SPRY1) expression (Shea et al., 2010). SPRY1 inhibits downstream effectors of the FGF signaling cascade, namely the ERK pathway, and fine-tunes the muscle stem cell response. *Spry1* expression is downregulated as the cells re-enter the cell cycle; however, re-activation of SPRY1 expression is required for cycling muscle stem cells to self-renew and return to quiescence.

Concurrently, Notch signalling is required to maintain muscle stem cell quiescence (Bjornson et al., 2012) (see poster). Expression of *Notch1* and *Notch3* are maintained by the transcription factor FOXO3 (Gopinath et al., 2014). Activation of Notch by its ligands Dellta and/or Jagged, followed by the release of the notch intracellular domain (NICD) triggers the expression of genes that are bound by the transcription factor RBPJ. These genes include known Notch targets, such as *Hes1*, *Hes5*, *Hey* and *Heyl* (Wen et al., 2012). Together, they repress the expression of the myoblast determination protein MyoD and prevent cell cycle re-entry. Knockout of *Hey1* and *Heyl* leads to the precocious activation of MyoD and ki67 in resting satellite cells (Fukada et al., 2011). Interestingly, Notch activation also increases the expression of *Pax7* through an upstream RBPJ binding site (Wen et al., 2012). This suggests that Notch signalling is essential to maintain the quiescent muscle stem cell state.

MicroRNAs (miRNAs) also play a main role in regulating muscle stem cell quiescence (see poster). Satellite cells that lack the miRNA-processing endoribonuclease DICER1 quickly lose quiescence and fail to correctly regenerate muscle upon injury (Cheung et al., 2012). Specifically, miRNAs such as miR-489 target the mRNA of cell cycle genes, in this case of the oncogene *Dek*, and prevent cell cycle re-entry. Another way by which miRNAs maintain the identity of satellite cell is by targeting the mRNA of myogenic factors. miR-31 targets the 3′UTR of *Myf5*, but does not lead to its degradation (Crist et al., 2012). Instead, the transcribed *Myf5* mRNA is stored in messenger ribonucleoprotein particle (mRNP) granules until satellite cells re-enter the cell cycle. This prevents the precocious expression of MYF5, and also increases the speed of activation and myogenic progression.

Together, these mechanisms allow quiescent muscle stem cells to remain relatively unchanged for extended periods. Moreover, they clearly demonstrate that the quiescent state is actively regulated and exists in a careful balance of opposing cellular pathways that prime the muscle stem cell for action.

## Maintenance and self-renewal of muscle stem cells

The total number of satellite cells in the tissue is maintained at homeostatic levels. Recent analysis of regeneration in muscles that are depleted of satellite cells have not only confirmed their essential requirement in tissue repair but also that its population is self-renewing (Lepper et al., 2011; McCarthy et al., 2011; Murphy et al., 2011; Sambasivan et al., 2011). In these experiments, no other cell type was able to re-establish the satellite cell pool when PAX7-expressing cells were ablated. This reaffirmed the notion that self-renewing muscle stem cells exist within the pool of satellite cells.

Upon activation, muscle stem cells are able to undergo asymmetric cell division to generate committed progeny without losing their own stem cell identity (Kuang et al., 2007; Rocheteau et al., 2012; Shinin et al., 2006; Troy et al., 2012) (see poster). This allows the number of stem cells to be maintained throughout regeneration. Although specific molecular triggers have yet to be identified, distinct components that make up the apical and basal surface of the muscle stem cell niche provides the stem cell with polarity cues (Kuang et al., 2008). Establishment of the mitotic spindle along this axis and activation of the PAR polarity complex segregates the phosphorylated form of the cell-fate determinants MAPK14 and MAPK11 (hereafter referred to as p38MAPK) (Troy et al., 2012). The daughter cell that contains p38MAPK commits to the myogenic fate and expands further to generate additional myogenic progenitors.

The asymmetrically divided daughter cell and the mother stem cell reciprocate feedback signals to further ensure their opposing fates. The Notch ligand Delta-like protein 1 (DLL1) is expressed by the committed progenitor to promote quiescence for the stem cell (Kuang et al., 2007). Inhibition of this signal by the γ-secretase inhibitor DAPT results in the loss of the stem cell population.

Muscle stem cells also have the capacity to symmetrically expand to replenish the stem cell pool (see poster). This mechanism maintains homeostatic numbers of satellite cells through rounds of injury and regeneration. The wingless family member Wnt7a that is secreted by regenerating myofibres activates the planar cell polarity pathway through the frizzled class receptor 7 (FZD7) and SDC4 receptor (FZD7−SDC4) complex (Bentzinger et al., 2013; Le Grand et al., 2009). Through the activation of the small Rho-GTPase Rac1, the stem cell division is polarised along the host myofibre, leading to a symmetric division that generates two identical stem cells. Consistently, *ex vivo* treatment of muscle stem cells with Wnt7a followed by transplantation results in higher numbers of engrafted satellite cells (Bentzinger et al., 2014). Moreover, the balance between symmetric and asymmetric cell division is carefully regulated by additional feedback from the regenerating niche. Transient expression of fibronectin by committed myogenic progenitors and resident fibrogenic cell types enhance Wnt7a-driven symmetric expansion (Bentzinger et al., 2013). Thus, muscle stem cells favour symmetric modes of self-renewal when tissue regeneration is well underway.

## Myogenic commitment

Commitment and differentiation to a myogenic fate requires the progressive expression of the myogenic regulatory factors MYF5, MYOD1 and myogenin (MYOG) (reviewed in Bentzinger et al., 2012; Moncaut et al., 2013). Since MYF5^+^ satellite cells and MYOD1^+^ myoblasts have poor engraftment potential and lack self-renewal capacity, MYF5 and MYOD1</emph> are considered determination factors. Committed MYF5^+^ MYOD1^+^ progenitors will undergo several rounds of cell division, before they terminally differentiate. The terminal differentiation of myogenic progenitors is marked by the early expression of MYOG and the subsequent expression of myofibrillar proteins, such as myosin heavy chain, just prior to fusion into myotubes.

Upregulation of MYF5 in muscle stem cells requires its transcriptional activation through the binding of PAX7 at the *Myf5* enhancer elements that are located 57 kb and 111 kb upstream of the transcription start site (McKinnell et al., 2008; Punch et al., 2009). In activated satellite cells, CARM1 phosphorylates the N-terminal arginine residues of PAX7, which leads to recruitment of the ASH2L−MLL histone methyltransferase complex to these enhancers, thereby driving the expression of *Myf5* (Kawabe et al., 2012). This mechanism is required such that asymmetrically divided muscle stem cells can commit to the myogenic fate. Cells that lack CARM1 are restricted to the primitive cell state and do not participate in proper tissue regeneration.

Several mechanisms regulate the expression and activity of MYOD1 in satellite cells, including the removal of transcriptional inhibition through the downregulation of HES and/or HEY (see poster panel ‘The quiescent muscle stem cell’). *Myod1* expression is triggered through FGF2 signalling, which induces downstream phosphorylation of p38MAPK (Jones et al., 2005) (see poster panel ‘Myogenic commitment’). This pathway is also implicated in fate determination after asymmetric cell divisions (Troy et al., 2012). Notably, MYOD1 has functional roles in the transcriptional activation of both proliferation and differentiation genes. In proliferative states, expression of differentiation genes is repressed by competitive binding of SNAI1 and/or SNAI2, and the recruitment of histone deacetylases to MYOD1-binding sites (Soleimani et al., 2012). MYOD1 also leads to the expression of miR-206, which targets the mRNAs of *Pax7*, *Snai1* and *Snai2* for degradation. This, temporally, regulates the transition from progenitor expansion to terminal differentiation. p38MAPK also phosphorylates several proteins, such as the E-protein E12/E47 (also known as TCF3) and the myocyte-specific enhancer factors 2 (MEF2s, i.e. MEF2A, MEF2B, MEF2C and MEF2D) that assemble on the *Myog* promoter to allow transcription. (Faralli and Dilworth, 2012). The interaction of MYOD1 with this transcription factor complex is important to induce gene expression of *Myog* and, consequently, activate other muscle-specific genes.

## Therapeutic approaches

Muscle stem cells have become the main therapeutic target in the treatment of muscle disorders. Boosting the regenerative capacity of diseased muscle has the ability to speed up tissue repair in acute injuries and to hinder the progression of chronic muscle diseases. Concurrently, both pharmacological and cell-based therapies are being developed. Even though pharmacological stimulation of intrinsic repair does not remedy inherent genetic defects, these routes of therapy usually reach the clinic faster, and do not face the level of economic and regulatory scrutiny that plague genetic or cell-based therapies (reviewed in Wang et al., 2013). Small-molecule drugs can be delivered systematically, thus acting on muscles such as the diaphragm that are ill-suited for cell injection. Most notably, the use of anti-inflammatory and anti-fibrosis drugs in muscular dystrophy normalises the muscle stem cell niche and stimulates regeneration (Mann et al., 2011; Manzur et al., 1996). Combined with the use of assistive devices and physiotherapy, this is currently considered the standard of care for muscular dystrophy patients. With continuing efforts towards understanding the regulation of muscle stem cells, new generations of pharmacological agents will be able to act with better specificity and effectiveness towards augmenting the kinetics of muscle regeneration.

Muscle stem cell transplantation holds great therapeutic potential because it could provide the host muscles of muscular dystrophy patients with a healthy genetic complementation and might increase the myogenic pool. However, the collection of sufficient material for transplantation makes this approach technically unachievable. Maintaining muscle stem cell stemness *in vitro* by modifying their microenvironment is a promising therapeutic avenue. It was shown that supplementation of substrates and/or extracellular matrix (ECM) proteins, such as fibronectin or collagen VI, can be used to mimic the satellite cell niche *in vitro*, and to improve the success of satellite cell self-renewal and transplantation (Bentzinger et al., 2013; Gilbert et al., 2010; Urciuolo et al., 2013). Another means to circumvent this problem is to isolate viable muscle stem cells from skeletal muscles several days post-mortem (Latil et al., 2012). Indeed, muscle stem cells are enriched in post-mortem tissue because of their quiescent state that confers a survival advantage in hypoxic environment.

Other major technical limitations of transplantation therapies are low cell survival and poor migration potential. Overexpression of pro-migration factors, such as VEGF or MMP9, or co-injection of myogenic cells with growth factors IGF1 or FGF2, showed potential to increase cell survival or migration but the effect on muscle function was limited or unknown (Bouchentouf et al., 2008; Lafreniere et al., 2009; Morgan et al., 2010). Interestingly, we could recently show that treating freshly isolated satellite cells with Wnt7a for a few hours *ex vivo* increased their migration potential *in vivo* by stimulating the planar cell polarity pathway (Bentzinger et al., 2014). This *ex vivo* stimulation resulted in a massive increase in the number of engrafted myofibres and had a hypertrophic effect on the engrafted myofibres, resulting in higher muscle force. Another promising therapeutic avenue comes from the conditional expression of PAX3 and or PAX7 in embryonic or induced-pluripotent stem cells (Darabi et al., 2011). These cells can be easily sourced and will engraft in the satellite cell niche through systemic injections. However, the use of embryonic stem-cell-derived therapies has not reached the clinic and will require in-depth safety trials. These results underline the importance of targeting specific mechanisms of stem cell behaviour in order to boost long-term engraftment and to increase the efficiency of cell therapies.

## Conclusions and perspectives

It has finally been realised that muscle stem cells can be used to recapitulate long-term regeneration of muscle cells. Recent molecular and genetic studies have furthered our ability to isolate muscle stem cell populations and modulate their behaviour *in vivo*. These important insights will facilitate the development of new therapies to expand muscle stem cells *in vitro* for transplantation and to enhance the regenerative capacity of damaged muscles. Eventually, these forthcoming cell-based therapies will allow for the generation of functionally stable muscle to correct genetic disorders in patients. Moreover, the understanding of cellular quiescence and activation is integral to regenerative medicine and may be applicable to other adult stem cell types. Taken together, future studies of muscle stem cells are imperative to gain a holistic understanding of adult myogenesis.

## Supplementary Material

Article Series
