# Supplementary material for: Muscle stem cells at a glance
Source: J Cell Sci. 2014 Nov 1;127(21):4543–8. doi: 10.1242/jcs.151209 (PMC4215708; doi:10.1242/jcs.151209)
Supplement: Article Series [file supp_127_21_4543_v2_index.html]

Article Series 

# Muscle stem cells at a glance

## Article Series
